# Social and Communicative Functions of Informed Consent Forms in East Asia and Beyond

**DOI:** 10.3389/fgene.2017.00099

**Published:** 2017-07-20

**Authors:** Go Yoshizawa, Teguh H. Sasongko, Chih-Hsing Ho, Kazuto Kato

**Affiliations:** ^1^Graduate School of Medicine, Osaka University Suita, Japan; ^2^Division of Human Biology, School of Medicine, International Medical University Kuala Lumpur, Malaysia; ^3^Human Research Ethics Committee, Universiti Sains Malaysia Health Campus Kubang Kerian, Malaysia; ^4^Institute of European and American Studies, Academia Sinica Taipei, Taiwan

**Keywords:** medical genomics, consent documents, group consent, family consent, community engagement

## Abstract

The recent research and technology development in medical genomics has raised new issues that are profoundly different from those encountered in traditional clinical research for which informed consent was developed. Global initiatives for international collaboration and public participation in genomics research now face an increasing demand for new forms of informed consent which reflect local contexts. This article analyzes informed consent forms (ICFs) for genomic research formulated by four selected research programs and institutes in East Asia – the Medical Genome Science Program in Japan, Universiti Sains Malaysia Human Research Ethics Committee in Malaysia, and the Taiwan Biobank and the Taipei Medical University- Joint Institutional Review Board in Taiwan. The comparative text analysis highlights East Asian contexts as distinct from other regions by identifying communicative and social functions of consent forms. The communicative functions include re-contact options and offering interactive support for research participants, and setting opportunities for family or community engagement in the consent process. This implies that informed consent cannot be validated solely with the completion of a consent form at the initial stage of the research, and informed consent templates can facilitate interactions between researchers and participants through (even before and after) the research process. The social functions consist of informing participants of possible social risks that include genetic discrimination, sample and data sharing, and highlighting the role of ethics committees. Although international ethics harmonization and the subsequent coordination of consent forms may be necessary to maintain the quality and consistency of consent process for data-intensive international research, it is also worth paying more attention to the local values and different settings that exist where research participants are situated for research in medical genomics. More than simply tools to gain consent from research participants, ICFs function rather as a device of social communication between research communities and civic communities in liaison with intermediary agents like ethics committees, genetic counselors, and public biobanks and databases.

## Introduction

The rapid development of research and technology in medical genomics over the last decade has raised new issues concerning research design, sharing and future use of samples or data, social risks, return of results, among others—all of which are profoundly different from those encountered in traditional clinical research for which informed consent was developed (Beskow et al., 2001; Mascalzoni et al., 2008; McGuire et al., 2008; Facio et al., 2012; Ayuso et al., 2013; Appelbaum et al., 2014b; Khan et al., 2014; Grady, 2015). Global initiatives for international collaboration and public participation in genomic science and technology now face increasing demand for international ethics harmonization to better assess the impact and dynamics of global genome research in the requirements of informed consent. As a result, research and policy experts in East Asia have been quick to follow and embrace such Western-oriented concepts and practices. This has created a growing tension between these concepts and those that arise from indigenous values, for which informed consent has been adapted to local contexts (Hull et al., 2004; Mascalzoni et al., 2010; Rotimi and Marshall, 2010; Henderson et al., 2014; Serepkaite et al., 2014; Yoshizawa et al., 2014).

This article focuses on informed consent documents for genomic research formulated for selected research programs and institutes from Japan, Malaysia, and Taiwan. This selection is seriously underrepresented even within their home countries. Rather than attempt to make broad generalizations about the situation in ‘East Asia,’ this article aims to explore the wide variety of perspectives and practices related to informed consent in the region. It finally extracts social and communicative functions from the consent forms and discusses implications of how and why these forms differ from the one that has been standardized in the West.

This study used a qualitative method for text analysis of four ICFs in three countries officially written in Japanese (Japan), English and Malay (Malaysia) and Chinese (Taiwan). First, these original documents were examined and summarized by section in English by each of the authors (GY, THS, and C-HH). Second, these English summaries were compared section-by-section between the three cases and consent documents in other regions including Europe, United States and Africa, supported by a vast literature on informed consent. Third, key sections and words for East Asian contexts were identified and categorized into two thematic domains related to communication and society.

## Japan

### Human Genomics and Informed Consent Process

In Japan many research institutions have now conducted human genome studies and several organizations manage large-scale biobanks including Biobank Japan (Triendl, 2003; Nakamura, 2007), the National Center Biobank Network (National Center Biobank Network [NCBN], 2017), and the ToMMo (Matsui and Tashiro, 2014). National databases include the Japanese Genotype-phenotype Archive (JGA), which is conducted in partnership with the National Bioscience Database Center (NBDC) and the DNA Data Bank of Japan (DDBJ) (Kodama et al., 2015), and the integrative Japanese Genome Variation Database (iJGVD) as partly supported by the ToMMo Project (Yamaguchi-Kabata et al., 2015). Human genome research is regulated by the Ethical Guidelines for Human Genome/Gene Analysis Research (‘Genome Guidelines’), which were established by three government ministries in 2001 and have been revised several times. The guidelines permit researchers to obtain broad consent, whereby informed consent is granted not only for a specific or defined project, but also extends to other genome analyses or to other related medical research (Porter, 2009). Initially in the 1990s, the Japanese legal concept of informed consent was quite similar to the concept formulated in Western law (Tejima, 2002), which has since been gradually and cautiously accepted into medical practice (Leflar, 2001). Japan’s health care policy is the first in the world to reimburse medical institutions for the process of informed consent (Akabayashi and Fetters, 2000). At the same time, informed consent activities have become more demanding and costly for professionals, allowing little time for patients to consider the implications (Sakaguchi and Maeda, 2005; Fukuda et al., 2009).

### Medical Genome Science Program (MGSP)

Medical Genome Science Program is one of the largest genomics projects in Japan and it has been funded by the MEXT since 1990. This project acted in concert with the development of the Human Genome Project, and it also gradually widened its research scope to include plant and animal genomes and bioinformatics, as well as the relationship between genome research and society (Itoh and Kato, 2005). In the fifth stage of this project, the GSP started to support MEXT-funded researchers in 2010. The MGSP is one of four sub-activities in the GSP and has conducted new-generation sequencing and associated bioinformatics analysis. The Research Unit for the ELSI of Genomics based in the GSP created an ICF for MGSP. The Unit drafted a template by reviewing a number of consent documents established in the country and abroad and following Japanese government guidelines and international norms and guidelines. The draft template was refined over 2 months of close discussion and communication between the Unit and executive researchers in MGSP and GSP. This interactive process made genome researchers aware of ethical implications of personal genome research, and, on the other hand, enabled ELSI researchers to incorporate pragmatic aspects of research into the design of the ICF (Minari et al., 2014a).

### The ICF of MGSP

The ICF template for 1st March 2013 consists of three parts: the information sheet, the consent certificate and the withdrawal of consent certificate. The information sheet is divided into introductory remarks and 13 sections. The sheet enumerates medical and scientific benefits as well as potential physical and psychological risks of the research. To ease research participants’ anxiety and concern, it stresses that researchers can introduce genetic counselors. There is an optional section for return of results where the participant may be able to receive incidental findings and the right not to know is also assured by checking the box in the consent certificate. Note that the ICF explains that the registration of research data to public databases enables a wider range of researchers to use the data and contribute to advances in medical science. There are two types of database access: those open to the public; and those limited to qualified researchers in aspects of scientific quality and managerial relevance to their activities. The information sheet also states that intellectual property rights as a result of genetic analysis shall belong to researchers or organizations, but not to participants. The consent certificate contains 13 boxes by section in the information sheet, to be checked by each research participant if they understand the content referred to. There are two indications of intention: one is whether to receive the results of genetic analysis; and the other is whether to agree with the storage of samples or information longer than the initially scheduled period of research, in the case of the extension or renewal of research. The research participant or his/her legal representative should sign the certificate with the date of signature. The withdrawal of consent certificate simply states that the research participant will withdraw his or her consent and requires their signature and the date of signature.

The end of the research may be extended or renewed with the approval of ERCs. In the case of withdrawal, research participants need to sign and submit the withdrawal of consent certificate (the place of submission is open to users of the template), and then his or her sample or information will be disposed of. However, the information sheet adds a disclaimer that this may not be possible once the information is published in academia or registered in public databases.

### Contextual Features

There are some specific features in the above ICF reflecting academic, legal and social contexts in Japan. Because this template is designed for the government-funded MGSP and the government has been developing national genome databases and biobanks, the value of the registration of data and samples to public databases and biobanks is emphasized in two sections of the information sheet. The term ‘disclosure of the results of genetic analysis’ refers to terminology used in the revised Genome Guidelines 2013, which follows the Protection of Personal Information Act 2003 (Inoue and Tsuruyama, 2015). In legal terms, researchers should disclose the results of genetic analysis at request of the research participant, regardless of whether the participant can fully understand the meaning of the results. The Genome Guidelines and the subsequent ICF make a practical compromise on the disclosure by stating that the results can be disclosed only when proved to be beneficial for medical treatment. In this sense, such disclosure can be called ‘returning’ the results, but the template prefers ‘disclosure’ as a more legalistic term, which implies that the research results are fundamentally attributed to researchers, and not to participants (Masaki et al., 2014). Likewise, re-identifiable anonymization is literally ‘linkable anonymization’ as a legal term representing anonymized and coded information that is re-identifiable with a separately stored table linking personal information and the corresponding code.

The template makes researchers aware that individuals and institutions are supporting medical genome research in several ways, as the term ‘research participant’ is referred to literally as ‘research cooperator’ in Japanese. First, for the function of genetic counseling is described in the information sheet. Genetic counselors are accredited by the Japanese Society for Genetic Counseling and the Japan Society of Human Genetics since 2005 and there are 205 accredited counselors as of December 2016 (Yamamoto et al., 2009; Japanese Society for Genetic Counseling, 2017). Because most research institutes do not have a counseling system and most studies may not require genetic counseling, the counseling system is not specified in the template. Second, the Genome Guidelines have been continually revised since 2001 and researchers have increasingly complained about the complication of its detailed instructions. To combat this tendency, the latest version in 2013 has changed its strategy by giving leeway to ERCs. Although this template was finalized before the publication of the latest version of Genome Guidelines, the role of ERC is noted repeatedly in the information sheet. Since 2012, the Research Unit for the ELSI of Genomics has organized annual meetings for members of ERCs on genomic research based in different institutions across Japan. The aim is not to standardize the process and the format of informed consent, but to facilitate mutual learning by exchanging experiences and views on the practical application of consent forms, such as broad consent, informed assent, and multi-center collaborative studies.

## Malaysia

### Human Genomics and Informed Consent Process

There are at least four major developments in Malaysia that help to describe the country’s situation in biomedical research—and especially in genomic science. First is the establishment of TMC Project, initiated in 2005 by the Malaysian government and based in Universiti Kebangsaan Malaysia. TMC has recruited a total of at least 106,527 participants and each donated blood and urine (Jamal et al., 2015). Second is the MyHVP, which was established in 2010 and based in Universiti Sains Malaysia. It developed a specialized database of Malaysian genetic variations, termed the MyHVP database (MyHVPDb) (Halim-Fikri et al., 2015), which currently deposited 291,718 SNPs datasets of 103 Malay individuals (Nik Hassan et al., 2016). Third is support from the Malaysian government on industry-sponsored clinical trials, which has subsequently lead to increasing number of trials where pharmacogenomic analysis was added as optional sub-study (Economic Transformation Program–Healthcare Sector, 2010; Pharmaceutical Executive Editors, 2015; Clinical Research Malaysia, 2016). Fourth is development in the area of medical genetic services. Although clinical sequencing has not entered routine clinical applications, clinical services, diagnostic services and training programs for medical genetics, including that of genetic counselors (Lee and Thong, 2013), are operational. These are mainly catered by the three main public universities (Universiti Malaya, Universiti Sains Malaysia, and Universiti Kebangsaan Malaysia) and the national-referral Hospital Kuala Lumpur, all located in Peninsular Malaysia.

Informed consent requirements have been well-described in various guidelines adopted by Malaysian research institutions (Abdul Rahman et al., 2011; Medical Research and Ethics Committee [MREC], 2017). MMC Guidelines on Medical Genetics and Genetic Services (Malaysian Medical Council [MMC], 2006) suggested that a blanket informed consent is the most efficient approach for stored genetic materials. This is exactly following the WHO proposal on guiding the practice (Wertz et al., 2003). In a broader context of informed consent process in Malaysia, there is an observation of significant medical paternalism between medical doctors and patients (Che Ngah, 2005), although it should be careful to generalize this to research practice. Another important observation is that, in lawsuit cases, patients have shown a lack of understanding of the consent documents, while medical doctors tend to overlook the fact that signed consent forms as such do not prove the patients’ understanding of and consent for the issue in question. This becomes a relevant extension to the practice of consent taking in genomic research, when one considers the legal case of *Havasupai Tribe vs. the Arizona Board of Regents* that ended in out-of-court settlement (Sterling, 2011).

### Universiti Sains Malaysia Human Research Ethics Committee (USM-HREC)

Malaysian Medical Council [MMC] (2006) published its guidelines on medical genetics and genetic services. This was intended primarily to guide medical practitioners in their practices with regards to medical genetics and genetic testing. The guidelines also addressed research, banking of DNA specimens and management of existing patient registries relating to medical genetic services. The guidelines, however, do not comprehensively and specifically address issues arising from the latest genomic technologies which are increasingly in use by Malaysian researchers. Realizing this lack, USM-HREC initiated a formulation of guidelines and an informed consent template for genomic studies in 2012.

This initiative was part of a broader context of quality and capacity building initiatives mandated by the USM-RCMO to USM-HREC which aims at obtaining international recognition for its ethical review practices. USM-HREC adopted the guidelines and the informed consent template by the end of 2012. The template was adapted from the United States NHGRI informed consent template for genomic studies version 12.8.10 with their permission. In 2013, the newly adopted guidelines and informed consent template were presented to the FERCAP conference. In 2014, FERCAP awarded compliance recognition to the ethical review practices of USM-HREC. USM-HREC and the MREC-MOH subsequently started a discussion on the initiation of a national network of ERCs. In March 2015, both institutions organized the first meeting of the NERCIM in Kuala Lumpur attended by ethics committees nation-wide where USM-HREC’s guidelines and ICF template on genomic studies were first introduced into the national audience. The 2nd NERCIM meeting in October 2015 decided to make ethical review practices of genetic and genomic studies as one of its focuses where USM-HREC’s documents serve as a model for an ongoing national harmonization. The following month, the guidelines and ICF template were published (Sasongko et al., 2015).

### The ICF of USM-HREC

The USM-HREC ICF template for genomic studies consists of three parts: the research information, the research subject information and consent form, and the research subject material publication consent form. The research information part has at least 12 sections and the latter two parts provide rooms for signatures of research participants. By default, the USM-HREC ICF encourages researchers to return or disclose individual findings whenever the information benefits the participants. Judgment about existence of such benefits lies with the researcher under consultation with USM-HREC and mainly depends on clinical significance, namely whether the information shows sufficient analytic and clinical validity and potential health implications either to individual research participants or his/her family members. In case of incidental findings, however, the ICF offers options if they would like to be informed. The in-house guidelines related to the ICF (Sasongko et al., 2015) stipulate that it is the responsibility of the researchers to provide genetic counseling in order to deliver individual findings and to factor in the genetic counseling cost, if any, into their research budget. In most instances, this will be under the coverage of the national health system. It is apparent that management of findings in ICF of USM-HREC stands as a mode of providing benefits and reward for participating in the research besides ensuring that researchers would not keep clinically meaningful individual genetic information from participants when it is acquired during the course of the research. In this regard, the ICF provides assurance to the participants that if it becomes available through the research, their clinically meaningful individual genetic information will be returned to them.

In addition, the USM-HREC ICF provides assurance to participants about confidentiality through sections that explain protection of identifiable individual information and submission to public database. It also states that withdrawal can be done through destruction of the sample and data, although data destruction would become impossible after it has been submitted to public database. Besides, although researchers are basically expected to destroy the samples after a certain period of time (years) following completion of the study, they can offer an option to participants to allow further storage by describing possible future use of the sample. USM-HREC may or may not require re-consent if researchers intend to pursue a new study using the stored samples. Such exchange is further proposed by notifying contact details of researchers’ and USM-HREC secretariat, when participants would like to execute withdrawal and if they have queries pertaining to research-related matters or rights of participants.

### Contextual Features

From the historical point of view, the model ICF was a locally university-based initiative which was later taken up at the national level. During the drafting process, emphasis was given to sections concerning return of results, incidental findings and sample/data storage. Due to the high costs of genomic technologies, there have been growing concerns about Malaysian researchers collaborating with researchers from more established countries or with wider international research groups. This has also inclined the researchers to share their data, re-use the samples for future research and re-analyze the data for different research objectives. While data sharing has been generally regarded as spreading out the benefits on the use of the data to a wider community, it has also been seen as potentially sensitive to individual and community privacy where the samples were taken. In Malaysia, it would be interesting to see how the Personal Data Protection Act 2010 (Azmi, 2011) affects formulation of consent documents. The Act makes an exception on the condition that data processing is necessary ‘for medical purposes and is taken by’ healthcare professionals [Section 40(1)] (Zawawi and Azmi, 2014). In this regard, this exception requires valid medical reasons to be present. However, as the consent provides a provision on the return of research results through genetic counseling and the necessary medical follow up, the Act may have an impact on the practice of the consent implementation. Although doctors appear to be aware of informed consent, practices which encourage patient engagement in shared decision-making are rare (Ng et al., 2013). While the ICF template mentions provisions regarding follow-up testing and counseling sessions, it is noteworthy that not all types of the required confirmatory testing must be carried out within Malaysia. Researchers are expected, however, to assist participants in looking for such testing especially if it is not available in Malaysia.

### Care for Ethnic Minorities and Family Consent

It is of note that Malaysian aborigines, or Orang Asli in local term, have been relatively frequent subjects of research, including genetic research (Tuck-Po, 2011). JAKOA (Jabatan Kemajuan Orang Asli) has requested that all research pertaining to Orang Asli should put forward a prior proposal to this government agency (JAKOA, 2017). There has been no stipulation pertaining to the type of consent that should be used when it comes to biomedical research. However, a recent report on Orang Asli (Aghakhanian et al., 2015 and Hoh Boon Peng, personal communication, 9 June 2016) employing a genomic methodology revealed a practice whereby prior consultation was done to the community elders before field visits to the community. During the field visits, explanation on the study was given en masse. Following the explanation, individuals interested to participate would approach the research personnel where another explanation would be given and individual signatures on the consent document were collected. This study was approved by JAKOA, Monash University (Malaysia)’s REC as well as MREC-MOH.

USM Human Research Ethics Committee (USM/JEPEM/15110477) and MREC-MOH (NMRR-15-2273-28140) have recently approved a whole exome sequencing study protocol which uses a family consent procedure. The study plans to perform whole exome sequencing on family trios. The consent process involves family discussion conducted by the research personnel. Names of the participating family members will then be listed on the signature page. An adult of legal age (normally the father) who is also the participant will sign on behalf of those whose names are listed. In addition, the USM-HREC ICF recognizes the possibility of incidental findings of non-paternity and researcher’s responsibility on the management of the information. While it does not seem operational to put the clause in the ICF (option of whether to be informed of such findings), the research team decided to firstly perform the consent process to the mother. Only when the mother indicates consent, the process for the family consent ensues.

## Taiwan

### Human Genomics and Informed Consent Process

Taiwan’s genetics and genomics research is embedded in the broader context of the development of biotechnology in Taiwan. In order to upgrade the country’s capacity from a manufacturing economy to a knowledge-based economy, since 1982, the government first listed biotech as one of the eight key technologies to promote for Taiwan’s industry and placed life sciences at the center of the state’s high-tech development plan (Chiu, 2002). For nearly 30 years, the government nurtured life sciences research by launching national programs and forming institutes to provide an amicable environment to promote biotech development. In 2003, the GRC was established by the Academia Sinica, the highest academic institute in Taiwan, and about 2 years’ later, the government launched the Biomedical Technology Island Plan with the aim of further fostering Taiwan’s genomics research and associated clinical trials. An important sub-project under this grand umbrella scheme is to set up a large-scale population biobank to support health and medical research in Taiwan with particular attention to developing personalized medicine (Fan et al., 2008). Since the preparatory phases, one of the main issues discussed among stakeholders has been how to set up a suitable governance framework for national-scale biobank practices that need to address legal and ethical concerns of privacy and informed consent (Lin et al., 2011).

In 2010, the Legislative Yuan (Parliament) passed the draft of the HBMA as a statutory basis for the establishment and management of all types of biobanks in Taiwan (Fan and Lin, 2011). This legislation stipulates informed consent requirements for sample collection and has been regarded as a basic regulatory framework for designing consent template with regards to biobank-related genetic research. On the other hand, the HSRA in 2011 stipulates rules on obtaining consent as a legal safeguard for the protection of human research subjects. In practice, however, as the governance framework for ICFs on genomics is rather fragmented, each hospital or medical research center relies on its own in-house ICF approved by the IRB. This study takes both the Taiwan Biobank ICF and the TMU- Joint IRB as cases to illustrate informed consent practices for genomics research in Taiwan.

### The ICF of the Taiwan Biobank

The Taiwan Biobank, funded by the Ministry of Health and Welfare, is a prospective long-term cohort which aims at collecting 300,000 samples from healthy Taiwanese population (200,000 samples) and patients (100,000 samples) in order to study the causes of diseases, i.e., to find out the relationship between genes, environment, and common complex diseases. The pilot study was initiated in 2009, followed by the formal collection of samples and data started in 2012. The Taiwan Biobank ICF consists of three parts: the information sheet, the advanced explanation sheet, and the consent sheet. The information sheet provides a general introduction to the Taiwan Biobank and the recruitment procedure for a participant to further engage with the biobank at a sample collection site. The advanced explanation sheet consists of 13 sections describing matters related to biobanking activities in greater detail. Apart from enumerating the purpose, rationale, and the competent authority of the Taiwan Biobank in order to inform participants about the general background about biobanking, this sheet comprises several sections that are worth further discussion as below.

First, the ICF of the Taiwan Biobank is carefully designed to avoid therapeutic misconception, whereby participants attribute therapeutic intent to research procedures (Appelbaum et al., 1982). Such information delivery is important in the consent form, on which the possible risks of taking part in biobank research are required to be acknowledged fully to form an accountable communication between biobank operators and participants. On the Taiwan Biobank ICF, such risks include not only individual ones of psychological and physical levels but also social ones, such as data leakage and stigmatization even though the ICF has explained that the research results will be published as collective data with no individually identifiable information released.

Second, the Taiwan Biobank ICF illustrated that a participant can request by written declaration to cease providing samples and data to the biobank, change the scope of the consented use, and withdraw from participation of the biobank at any time. It also outlines options for participants to decide if and under what conditions that they would be willing to be re-contacted by the biobank operator. Obtaining consent is treated as a long-term practice rather than a once-time commitment so that participants are given opportunities to change their minds whenever they wish. Furthermore, according to the benefit sharing requirement stipulated by Article 21 of HBMA in Taiwan, any profits derived from commercial use and received by an operator or the Biobank shall be given back to the human population groups or specific population groups to which the respective participants belong. This benefit sharing clause has been integrated into the Taiwan Biobank ICF. Even though the ICF articulates that biobank participants are not entitled to claim any property rights derived from the biobank research as such and that this property belongs to the research entity, it nevertheless specifies that the participants are entitled to share benefits according to the stipulations of HBMA. For this it also states that participants can contact the biobank operator or the EGC of the Taiwan Biobank about their complaints and infringement concerns. The EGC functions not only as a contact point, but also an independent review institution. As the biobank requires broad consent, it is up to the scrutiny of the EGC to further decide if an unspecified research purpose in the future will be approved.

### The ICF of TMU-Joint IRB on Genomics Research

This ICF template consists of both an explanation sheet and a consent sheet. The explanation part is further divided into 16 sections. Like the Taiwan Biobank ICF, it also requires PI to illustrate the purpose of the study and suggests that the target genes for research need to be described as specifically as possible and identifies the potential physiological, psychological, and social risks for participants to take part in the genomic research. However, unlike biobank-related research, the ICF on genomics studies requires PI to disclose identities of those who will access to samples and data and if the samples will be transferred to other research entities outside of Taiwan (and if so, it shall also indicate the related personnel). As for the secondary use of samples and data, the ICF allows participants an option to further decide if they would be willing to have their samples to be used for different biomedical research purposes (in such cases, another ICF is required to be signed) or if instead they would prefer their samples to be disposed of after the completion of the current research.

In terms of research feedback, participants are allowed to decide if they would like PI to inform them of research results or provide related medical information and counseling services. If not, the ICF suggests that PI needs to state clearly that the individual research results will not be provided even though participants are still welcome to access to the research results that have been published as an aggregated data. Furthermore, the ICF also allows participants to decide how they would like their samples and data to be handled if later on they decide to withdraw from the study. Such options for participants include the following—either samples or data can be used for the current study unless otherwise indicated, or samples and data will have to be disposed of immediately when participants withdraw from the current research. According to the ICF of the TMU-Joint IRB, there is a requirement for PI to notify participants of any derived rights and benefits, including commercial benefits according to the rules of HSRA. Moreover, the ICF explains the protection of genetic information through anonymization and the aggregation of personal data. However, it does not exclude the possibility of releasing the data to a competent authority for investigation according to law, and that is quite different from the ICF used for biobank-related research stipulated by HBMA. The ICF also provides contact information of its IRB for participants who need further information.

### Contextual Features

The above case study demonstrates that the informed consent practices for genomic studies in Taiwan vary widely. Apart from the biobank-related study and the Taiwan Biobank, which are mainly regulated by HBMA, research centers and hospitals rely on their in-house ICF templates that follow HSRA to obtain consent for genomics research. One example of this variety lies in whether participants’ data and information can be released and used for non-research investigation purposes. Such use of the data outside of the original purpose of research has been explicitly prohibited by HBMA (Article 20), which applies to all biobank-related research, but it may be permitted by an in-house ICF template for genomic studies as the TMU case demonstrates. Efforts toward standardization of various consent forms for genomics research have not been successfully implemented even though Taiwan is a member of FERCAP network. Thus even though the TMU case could not be regarded as a representative, it demonstrates an example of diverse consent practices in Taiwan. Furthermore, while genetic counseling services have developed in recent years (Chien et al., 2013), whether each service is required to return research results—especially regarding incidental findings—or whether they have to provide genetic counseling may also vary by consent form and the nature of the biomedical research in question.

### Group Consent

Except for the individual consent, there is also a statutory obligation for collective consent when research subjects involve Taiwanese aborigines. According to the Taiwan Aboriginal Basic Law (Article 21) and HSRA (Article 15), when the research subject involves aboriginal people, which are approximately of 2% of the whole 23 million populations in Taiwan, consultations need to be carried out for obtaining collective consent from the aboriginal groups. Such group consent is required not only for biomedical research itself but also for publication of research results about Taiwanese aborigines. To date there have been debates and discussions on drafting implementing rules of group consent that are to be proposed from the Council of Indigenous People – the competent authority of aboriginal matters in the Cabinet (Executive Yuan). The practice of informed consent in Taiwan has demonstrated that individual consent may not be sufficient for genomics research when the research subjects are ethnic minority groups, and that the publication of research results may have unfavorable effects—such as stigmatization—for these particular groups (Ho, 2012; Lin and Liao, 2012; Munsterhjelm, 2014).

## Comparative Analysis

This analysis not only compares the above ICFs, but also examines ICFs from other regional contexts. In Europe, the European Union (EU) Clinical Trial Directive (2001/20/EC) defines informed consent and contains guidance on how to draft a consent form. With respect to human genomics, there is a study of 14 consent form documents from 2004 to 2010 used in European GWASs projects (Boddington et al., 2011). A more recent study examines consent forms of Genomics England’s 100,000 Genomes Project (Chow-White et al., 2015). H3Africa Working Group on Ethics developed a set of guidelines for informed consent on genomic research in 2013, and a recent study analyzed informed consent documents for 13 of the 19 H3Africa projects (Munung et al., 2016). There seem no comparable review studies in the United States, but the informed consent elements are required by the Common Rule (45 CFR 46, Subpart A) and sample documents are provided by NHGRI. International documents include an ICF template for the 1000 Genomes Project (2016) and the ICGC model consent brochure (International Cancer Genome Consortium [ICGC], 2010). These references are qualitatively analyzed and compared with the above documents on a section or clause basis by taking into account different policy, social and cultural contexts. The comparative text analysis identifies communicative and social functions of the ICF, which may highlight East Asian contexts as distinct from other regions (**Table 1**).

**Table 1:** Statement on social and communicative functions in the ICF.

Communicative functions	MGSP, Japan	USM-HREC, Malaysia	Taiwan biobank	TMU-Joint IRB, Taiwan
Re-contact options	Incidental findings (Section 5)	Incidental findings (Section 7); Future use of specimens (Section 12)	Follow-up (Section 7)	Future use of specimens (Section 8); Change of the scope (Section 14)
Interactive support	Reference to genetic counselors (Section 11)	Independent opinion on the research and the rights of participants (Section 9)	Appeal about participant’s complaints and infringement concerns (Section 13)	Contact information of the IRB (Section 16)
Family or community engagement	n/s	Family consent allowed; Community consultation required for Malaysian Orang Asli	Group consent for Taiwanese aborigines would be required (under consultation for its implementing rules)	
**Social functions**				
Genetic discrimination	Social discrimination (Section 4)	Distress to participants and their families (Section 5)	Risk of stigmatization (Section 10)	Impact on social interests in education, employment and medical care (Section 4)
Sample and data sharing	Public storage of samples and data for medical advancement (Sections 7, 8)	Submission to a nationally or internationally shared database as public data release (Section 11)	Data and information can be linked to national databases with the approval from the EGC (Section 9)	n/s
Role of ethics committee	At the extension of research duration and initiation of new research (Section 9)	Independent opinion; Contact point (Section 9)	Independent review institution; Contact point (Section 13)	Independent review institution; Contact point (Section 15)

### Communicative Functions

Improvement of participants’ literacy is a critical challenge to achieving valid consent (Raich et al., 2001; de Vries et al., 2011), which also requires the improvement of readability and content in ICFs (Jefford and Moore, 2008; Nair and Ibrahim, 2015). Readability of the informed consent document has been a major discussion topic in research and practice, but the above case study demonstrates three other communicative functions of ICFs which have been relatively overlooked in early literature – namely, re-contact options, interactive support, and family or community engagement.

First, each ICF sets several options for participants regarding the future use of data and samples, how to withdraw from research and whether they wish to be re-contacted with regard to incidental findings. Re-contact options on incidental findings are explicitly stated in Malaysian USM-HREC and suggested in Japanese MGSP. This is a staged consent model, in which participants are given time for consideration in order to come to understanding decisions (Bunnik et al., 2013), particularly in the ICF that they receive information about incidental findings later when reported (Appelbaum et al., 2014a). In the United States, the American College of Medical Genetics and Genomics (ACMG) recommended re-contact policy settings for informed consent on new knowledge about the significance of particular results of genome/exome sequencing (ACMG Board of Directors, 2013). Re-contact options for future use of data and samples are set in the ICF of TMU-Joint IRB in Taiwan, as sometimes stated in European and African context (Boddington et al., 2011; Munung et al., 2016). In contrast, re-contact options in Taiwan Biobank are mainly set for follow-up purposes. A Japanese study on biobank research participants reveals that post-consent communication serves as a trust building experience (Watanabe et al., 2011), so re-contact options may give another opportunity to facilitate meaningful communication between researchers and participants.

Second, the above forms offer counseling or consultation support for research participants, following government guidelines in Japan or the practice of the plurality of opinions in Malaysia. In Taiwan, such genetic counseling service does not appear on the Taiwan Biobank consent form, but participants can appeal to the biobank or the EGC about their complaints and infringement concerns. While there appear no similar statements on counseling or consultation support in other continents, a Canadian study reveals that the majority of consent documents did not mention genetic counseling (Egalite et al., 2014). Such intermediary services become crucial as the boundary between research and treatment become increasingly blurred and therapeutic misconception can be fueled by participants and researchers (Halverson and Ross, 2012; Tupasela et al., 2017).

Third, informed consent forms function as a communication tool not merely between researchers and participants. In the process of informed consent on clinical decision-making, family members also have a role for decision support and anxiety reduction in Japan (Hattori et al., 1991; Ruhnke et al., 2000; Sullivan, 2015), while in Taiwan they act as liaisons for information and advocates in the physician-patient relationship under the legal framework (Lin et al., 2013). With a growing need for family centric initiatives for obtaining consent in genomics research (Minari et al., 2014b), East Asian countries may be quick to absorb a new model of informed consent, as the above Malaysian ICF suggests. Underlining communicative functions of consent forms also allows researchers to collaborate with others in a public domain and to empower their community (Chokshi et al., 2007). In Taiwan, HSRA stipulates group consent requirements when Taiwanese aborigines are recruited as research subjects in genetics and genomics research. This reflexively nudges participants to pay attention to the potential benefits and risks of genetic analysis for their ethnic communities. It is too simplistic to say that ethical decision-making in East Asian countries is more family- or community-centered than Western countries (Akabayashi and Slingsby, 2006; Chen et al., 2013), but it is nevertheless true that family and community considerations are important in clinical practice and research in East Asia (Pratt et al., 2014). Like in Africa (Ramsay et al., 2014), community approval would not supersede individual informed consent in East Asia. As a pre-research community engagement process is recommendable to develop consent form for genetic research (Marsh et al., 2010; Brief et al., 2012), systematic documentation of community engagement would become a key part in the consent process in Malaysia (Allotey et al., 2014). ICFs thus illuminate the relevance of communication between researchers and participants through research projects and the value of communities engaged with participants (Skinner et al., 2015).

All the above three communicative functions imply that informed consent cannot be validated solely with the completion of a consent form at the initial stage of the research, and informed consent templates can facilitate interactions between researchers and participants through (even before and after) the research process.

### Social Functions

Informed consent forms can also shed light on social aspects of genomics research. There are at least three social functions identified from the above cases in comparison with documents in other regions; genetic discrimination, sample and data sharing, and the role of ethics committee (i.e., ERC, IRB, or REC).

First, while many research participants are afraid of the risks of genetic discrimination through participation in genetics research (Hamvas et al., 2004), most East Asian countries do not have legal safeguards against genetic discrimination (Joly et al., 2010; Yoon et al., 2011; Otlowski et al., 2012). However, as in the case of a Japanese genetic cohort study (Matsui et al., 2008), most research participants may wish to have future disclosure of individual risks. Against this backdrop, each of the above ICFs tries to deliberately mitigate participants’ anxiety about possible social risks not followed by current legislations. Similarly, H3Africa Guidelines include risks of group discrimination and stigmatization as suggested elements for genomic studies.

Second, the consent template for MGSP in Japan encourages researchers to register their data and samples to national public databases and biobanks. In Malaysia, researchers have come under pressures in global research collaborations, for which sharing data via national or international databases is motivated as in the USM-HREC consent form. In Taiwan, even though the feedback of incidental findings is not allowed in the practice of Taiwan Biobank, according to Article 21 of HBMA, participants are entitled to share the benefits from biobank research. Co-funded by the National Institute of Health and the Wellcome Trust, all H3Africa consent documents are required to comply with the American Common Rule and thus make mention of data sharing (H3Africa, 2013; Wright et al., 2013). In effect, most of them included a statement about data and sample sharing by reason of progression of research, best practice, or the right thing to do (Munung et al., 2016). On balance, a variety of guidelines and sample ICFs collected from all over the world reflect different stances in sample and data sharing between (1) Europe and North America, which have established internationally recognized and well-networked databases and biobanks; (2) Far East Asian countries, which have promoted state-sponsored databases and biobanks as national catching-up strategies (Kuo, 2011); and (3) South East Asia and Africa, which have begun to provide genome data and samples for global research and practice. In Africa, however, clear concerns about requesting consent for the wide sharing of data and samples are reported (Ramsay et al., 2014).

Third, in the United States, most chairs of IRBs at centers conducting genomic studies do not know what is required for local boards regarding informed consent (Simon et al., 2011), while a number of different domestic IRB collaborations are in place (Barchi et al., 2014). In South Asia, ERCs become incapable of protecting human subjects against competing social and national interests (Simpson et al., 2015). Going against the tendency of more emphasis on the importance of data in making and informing decisions in ethics committees (Sugarman, 2004), consent forms may need to be carefully designed to reflect social and national interests as well as local values placed by wider engagement so that the ethics committees can address and review issues more promptly and effectively. There are also differences in how to use consent documents between ERCs (Edwards et al., 2007). In developing countries, written consent was documented in most cases, but was more likely to be obtained by researchers and participants with higher literacy (Hyder and Wali, 2006). Under the circumstances, African experience suggests that IRB members should be trained on all phases of research methodology including informed consent (Falusi et al., 2007; Kaas et al., 2007). In Europe, harmonization between RECs has been driven initially by the Clinical Trials Directive (Hedgecoe et al., 2006), and also recently promoted by the European Forum for Good Clinical Practice (EFGCP) (Davies et al., 2009; Cairoli et al., 2012). Given no such directives or common rules regionally adopted throughout a continent except FERCAP, ERCs in East Asia are likely to have considerable discretion about how to document and review consent forms. In this sense, clear statements on the role of ERC in consent documents, as demonstrated in the above case study, would construct more dynamic and responsible relationship between the research participant and the ERC.

## Conclusion

The section-by-section text analysis of different ICFs for a variety of genomic studies in East Asia reveals critical features that reflect local contexts as distinct from consent documents formulated in other regions. In terms of communicative aspects, East Asian consent forms are keen to set plural options and contact points and in some cases facilitate family or community engagement for the research participant. From a social perspective, first, there are statements on potential social risks for research participants in the above ICFs. Because most East Asian countries do not have legal safeguards against genetic discrimination, the above consent forms are deliberately designed to warn participants about such social risks. Second, most of the above consent forms impress upon researchers and participants the relevance of data and samples, as well as the benefit of sharing via public databases or biobanks. Third, statements on the role of ethics committees suggest dynamic and responsible relationships between the research participant and the committee so that East Asian ethics committees are likely to have considerable discretion about documentation and review of ICFs. Thus consent forms stand not just as a legal contract between individual researchers and participants but rather as a device for social communication between research communities and civic communities in liaison with intermediary agents like ethics committees, genetic counselors, and public biobanks and databases. Although international ethics harmonization and the subsequent coordination of consent forms may be necessary to maintain the quality and consistency of the consent process for data-intensive international research (Dove et al., 2016), it is also worth paying more attention to the local values and different settings of the locations where medical genomic research is conducted.

## Author Contributions

THS drafted the section of Malaysia and helped to draft the manuscript. C-HH drafted the section of Taiwan and helped to draft the manuscript. KK conceived of the study and participated in its design and coordination. GY drafted the section of Japan, conducted the comparative analysis and edited the whole manuscript. All authors read and approved the final manuscript.

## Conflict of Interest Statement

The authors declare that the research was conducted in the absence of any commercial or financial relationships that could be construed as a potential conflict of interest.

## Abbreviations

EGC: Ethics and Governance Council of the Taiwan Biobank
ELSI: ethical, legal and social implications
ERC: ethics review committee
FERCAP: Forum for Ethical Review Committees in the Asian and Western Pacific Region
GRC: Genomics Research Centre, Taiwan
GSP: Genome Science Project, Japan
GWASs: genome-wide association studies
H3Africa: Human Heredity and Health in Africa
HBMA: Human Biobank Management Act, Taiwan
HSRA: Human Subjects Research Act, Taiwan
ICF: informed consent form
ICGC: International Cancer Genome Consortium
IRB: institutional review board
JAKOA: Department of Orang Asli Development, Malaysia
MEXT: Ministry of Education, Culture, Sports, Science and Technology, Japan
MGSP: Medical Genome Science Program, Japan
MMC: Malaysian Medical Council
MREC-MOH: Medical Research Ethics Committee of Malaysian Ministry of Health
MyHVP: Malaysian Node of the Human Variome Project
NERCIM: National Ethics Review Committees in Malaysia
NHGRI: National Human Genome Research Institute, United States
NHID: National Health Insurance Database, Taiwan
PI: principal investigator
REC: research ethics committee
TMC: The Malaysian Cohort
TMU: Taipei Medical University
ToMMo: Tohoku Medical Megabank, Japan
USM: Universiti Sains Malaysia
USM-HREC: USM Human Research Ethics Committee
USM-RCMO: USM Research Creativity and Management Office
WHO: World Health Organization.
